# A Sensibility Assessment of the Job Demands and Accommodation Planning Tool (JDAPT): A Tool to Help Workers with an Episodic Disability Plan Workplace Support

**DOI:** 10.1007/s10926-022-10057-4

**Published:** 2022-07-14

**Authors:** Monique A. M. Gignac, Julie Bowring, Sabrina Tonima, Renee-Louise Franche, Aaron Thompson, Arif Jetha, Peter M. Smith, Joy C. Macdermid, William S. Shaw, Dwayne Van Eerd, Dorcas E. Beaton, Emma Irvin, Emile Tompa, Ron Saunders

**Affiliations:** 1 Institute for Work and Health, 400 University Avenue, Suite 1800, Toronto, ON M5G 1S5 Canada; 2 Dalla Lana School of Public Health, University of Toronto, Toronto, ON Canada; 3 WorkSafeBC, Vancouver, BC Canada; 4 Workplace Safety and Insurance Board (WSIB), Toronto, ON Canada; 5 Faculty of Medicine, University of Toronto, Toronto, ON Canada; 6 School of Physical Therapy, Western University, London, ON Canada; 7 Medicine, University of Connecticut Health, Farmington, CT USA

**Keywords:** Disability, Employment, Support, Accommodations, Measurement

## Abstract

*Purpose* Sensibility refers to a tool’s comprehensiveness, understandability, relevance, feasibility, and length. It is used in the early development phase to begin assessing a new tool or intervention. This study examined the sensibility of the job demands and accommodation planning tool (JDAPT). The JDAPT identifies job demands related to physical, cognitive, interpersonal, and working conditions to better target strategies for workplace supports and accommodations aimed at assisting individuals with chronic health conditions. *Methods* Workers with a chronic health condition and workplace representatives were recruited from health charities, workplaces, and newsletters using convenience sampling. Cognitive interviews assessed the JDAPT’s sensibility. A 70% endorsement rate was the minimum level of acceptability for sensibility concepts. A short screening tool also was administered, and answers compared to the complete JDAPT. *Results* Participants were 46 workers and 23 organizational representatives (n = 69). Endorsements highly exceeded the 70% cut-off for understandability, relevance, and length. Congruence between screening questions and the complete JDAPT suggested both workers and organizational representatives overlooked job demands when completing the screener. Participants provided additional examples and three new items to improve comprehensiveness. The JDAPT was rated highly relevant and useful, although not always easy to complete for someone with an episodic condition. *Conclusions* This study highlights the need for tools that facilitate accommodations for workers with episodic disabilities and provides early evidence for the sensibility of the JDAPT.

## Introduction

Increasing numbers of people living with chronic health conditions are employed [1, 2]. New treatments and health care procedures have enabled individuals who were previously unable to work because of their health to find, sustain, or return to work (RTW) [3–7], while technological improvements, including more opportunities for remote working, can help to make employment more accessible for people living with chronic health conditions [8]. Frequently, the activity limitations experienced by people working with chronic conditions are episodic. That is, workers with a chronic condition report experiencing periods of wellness with no job limitations (i.e., no disability) punctuated by periods of more severe symptoms that contribute to an intermittent disability at work [9–12]. Examples of chronic conditions associated with episodic disability include mental health conditions like depression and anxiety, Crohn’s disease, colitis, multiple sclerosis, migraine, rheumatic diseases like arthritis and lupus, chronic fatigue syndrome, and many musculoskeletal conditions (e.g., low back pain, tendinopathies). Extended life expectancies for individuals living with conditions like HIV/AIDS and some types of cancer have meant that these conditions also are associated with episodic disabilities at work. Although different in their etiology, many conditions causing episodic disability have similar impacts on employment, with individuals finding it challenging to meet the demands of their job.

In addition to their episodic nature, the signs and symptoms of many chronic conditions are often “invisible” to others until a person experiences an episode or flare or chooses to disclose the condition. That is, others are often unaware that an individual is living with a chronic condition that can cause an episodic disability [10–15]. The episodic and invisible nature of many chronic health conditions can create challenges for workers in seeking and obtaining workplace support because of the uncertainty about whether to disclose their condition and, if disclosed, because of uncertainty about what type of information to share in the workplace. Disclosing personal health information may result in greater support, but there may be accompanying stigma and concerns about one’s future career [12, 15–27]. Not sharing information may protect a worker from stigma but may mean delays in receiving support until there is a crisis, fewer support options, and misinterpretations of workplace behaviours as reflecting a lack of motivation, skills, or as a negative interpersonal style [12, 14, 20, 28–30]. Moreover, research finds that reports of unmet accommodation needs are associated with greater workplace activity limitations, more job disruptions (arriving late/leaving early), and perceived productivity losses [6, 7, 10, 31–36].

### Job Demands and Accommodation Planning Tool (JDAPT)

Several measures and tools have been developed to address the complexity of disclosure decisions and provision of workplace support [37–43]. Legislation in many industrialized countries also provides regulations and protections to prevent discrimination and to promote the full participation in employment of people living with disabilities [44–47]. The legislation is often rooted in fundamental values of equality and diversity, which are increasingly being tackled by global bodies and individual workplaces [48]. For example, in Canada, laws protect personal health information and require that organizations make reasonable accommodations for workers living with disabilities [47, 49]. Workplaces are encouraged to focus on social and environmental barriers that can make employment difficult and not on medical diagnoses and symptoms. A variety of research tools exist to measure perceptions of work, including job demands and the psychosocial work environment. For example, the Work Ability Index is often used in clinical occupational health and workplace surveys to estimate current work ability, a workers health status and the potential need for future resources [50, 51]. Other research tools like the Job Content Questionnaire (JCQ) and the Copenhagen Psychosocial Questionnaire (COPSOQ) have been extensively used to assess a workers perceptions of their work environment [52, 53]. However, existing research tools do not help a worker think through the intermittent or ongoing challenges of their various job demands or provide strategies for self-management, informal and formal support relevant to each demand. A recent scan of publicly available self-help resources and supports found that many existing resources were disease-specific and focused largely on management of condition symptoms [43]. Resources often did not provide material focused on diverse job demands, and most were not interactive but provided general pros and cons related to decision making with limited information about informal and formal supports and accommodations [43].

To address these gaps, we created the JDAPT. The purpose of the JDAPT is to help a worker, or an organizational representative like a supervisor, identify key job demands at a broad level without having to discuss health diagnostic or symptom information. Respondents input information pertinent to their own or another’s job demands and indicate whether health issues create challenges in meeting job demands on an intermittent or ongoing basis. They are then provided with ideas for informal and formal self-management and supports tailored to the areas where they report difficulties. The JDAPT is intended to be flexible and relevant to a wide variety of health conditions, job types, and workplace contexts. It is broader than functional workplace assessments that focus primarily on the physical working demands of a job (e.g., repetition, load and time spent on tasks like standing and lifting) or cognitive demands assessments (e.g., memory, problem solving, executive functioning in tasks like concentration) by measuring job demands across four domains—the physical demands of a job, cognitive demands, interpersonal demands, and working conditions—which are then linked to examples of self management, support, and accommodations.

As a first step to evaluating the JDAPT, we wanted to assess the “sensibility” of the job demands questions. “Sensibility” is a term originally used by Feinstein [54] to highlight the necessity for new measures and tools in their early development phase to demonstrate fundamental attributes like comprehensiveness, understandability, relevance, and feasibility. Similar approaches have been emphasized in health care studies when examining the clinical utility, practicality, or applicability of a tool [55–58], as well as in research discussing content validity [59–61]. Essential to the process is a method that provides opportunities for the individuals who will ultimately use the measure or tool to provide their perspectives on whether the concepts and questions are meaningful to them and whether there are gaps and omissions in the tool. Irrespective of assessing the psychometric qualities and robustness of a tool, if it does not meet the needs of workers with chronic conditions, or if it is impractical to apply, it is unlikely to be the optimal choice for use in the workplace. The objective of this study was to test the sensibility of the JDAPT across a variety of chronic health conditions and job types and sectors as part of initial development testing. We assessed two versions of the JDAPT, one for workers and one for workplace representatives (e.g., supervisors, HR personnel, disability managers) who provide support to workers living with chronic conditions giving rise to episodic disabilities. We focused on the JDAPT’s comprehensiveness, understandability, relevance, feasibility, and length.

## Methods

### Participants

Study participants were workers living with diverse chronic health conditions, as well as organizational representatives, all of whom were recruited using convenience sampling. Recruitment took place from September 2019 to May 2020. We contacted Canadian health charities serving individuals living with physical or mental health conditions, workplace contacts established through the Institute for Work & Health, where the project is based, the research project website, and newsletters. Recruitment material informed potential worker participants that we were interested in interviewing individuals “currently employed and living with a chronic physical or mental health condition that can sometimes make working difficult.” Potential organizational representatives like supervisors, human resources professionals and disability managers were told we were interested in interviewing individuals “with experience supporting employees with chronic physical or mental health conditions.” Some materials stemming from health charities noted specific health conditions (e.g., multiple sclerosis, Crohn’s disease). Interested individuals contacted the study team by email or telephone. Eligible participants were employed, lived with a chronic physical or mental health condition causing an episodic disability (worker participants) or had workplace experience supporting workers with chronic conditions causing episodic disability (organizational participants). Participants were ≥ 18 years of age and able to complete an English-language interview. The research team worked to recruit a diverse sample in terms of gender, age, ethnic diversity, and occupational type (e.g., including non-office work). Participants received an honorarium of CAD $75.00. Informed consent was obtained from all participants. Ethics approval was received from the University of Toronto Research Ethics Board (#37970).

### Measure: Job Demands and Accommodation Planning Tool

The JDAPT identifies broad areas of work related to physical, cognitive, interpersonal (i.e., working with others) and working conditions where self-management, informal supports or formal accommodations may be useful in managing difficulties with job tasks, and where a worker might be able to avail themselves of supports without having to disclose a health diagnosis or disability type. Two versions of the JDAPT were developed: an *employee version* for someone with a chronic condition causing an episodic disability to complete, and an *organizational version*, for someone supporting an employee. The latter version could be completed by the organizational representative on their own or with the worker. The two user-versions were identical except for small changes in the instructions and in the framing of the questions (e.g., “your job” and “my health” in the employee version were replaced with “this employee’s job” and “their health” in the organizational version).

Prior to filling out the complete JDAPT questionnaire, a short number of screening questions were developed to potentially decrease the number of items completed as part of the full JDAPT. The screening questions would allow respondents to skip an entire domain of the JDAPT (e.g., physical demands) if the respondent reported that the domain was not relevant to their job, if they were not having any difficulties with the domain, or if they were not interested in learning more about supports relevant to the domain. Examples of job demands were provided for each screening domain that were similar to those for the specific job demands asked in the complete JDAPT. For the purposes of the current study, all participants completed both the screening questions and the complete JDAPT questionnaire regardless of their responses to the screening questions. This allowed us to examine concordance in the perceived relevance of JDAPT domains assessed through the screening questions with the complete assessment using the full JDAPT.

The complete JDAPT comprised 21 job demands (see Appendix A). Four items asked about the physical aspects of a job (e.g., working with your hands, physical endurance or stamina); six items asked about cognitive or “thinking” aspects of a job (e.g., paying attention to detail, concentrating for long periods of time, managing time pressures); four items asked about interpersonal demands or working with others (e.g., supervising others, dealing with distressed or angry people); and seven items asked about working conditions (e.g., working with hazardous equipment or situations, isolated work, changing schedules or shifts, working in situations where making an error has critical consequences). Multiple examples were provided to illustrate each job demand, and that considered different types of jobs and industrial sectors. Participants were first asked whether a particular job demand was important to their job. If they indicated that it was not an important part of their job, they moved to the next job demand. If an individual indicated that a job demand was important to their work, they were asked about their ability to meet the job demand (no difficulty, some difficulty, a lot of difficulty/unable to do) and whether their difficulty was stable or variable (no change over time, sometimes changes depending on their health, changes a lot because of their health). An opportunity to add additional job demands was provided after each of the four domains. Upon completion of the JDAPT, participants would normally be given a personalized report and ideas for a range of self management and work-related support and accommodation strategies that are tailored to each specific job demand. Because this study focused on examining the JDAPT questions themselves, participants did not receive a summary or support/accommodation ideas and strategies.

### Procedure and Sensibility Assessment:

Although the JDAPT was designed to be an online tool, for the purposes of sensibility testing, all participants were provided with a paper workbook which contained the screening questionnaire and job demands questions. Participants were interviewed face-to-face or by telephone. An in-depth cognitive interview was developed [58, 62–65]. As part of the interview, workers living with a chronic condition causing an episodic disability were asked to complete the questions for their current health and job demands. Organizational representatives were asked to think about a particular experience with a worker and complete the JDAPT with that person in mind.

Participants were asked to first complete the screening questions. The interviewer recorded the time to complete the items. After completing the screening questions, respondents were asked questions about its understandability, relevance, and feasibility. Table 1 provides an overview of the concepts assessed. Participants then filled out the complete JDAPT. Similar to the screening questionnaire, the interviewer recorded the time to finish the questions. This was followed by an in-depth cognitive interview that assessed overall comprehensiveness, understandability, feasibility, relevance, and perceived length, as well as questions about each item in the JDAPT and the examples and concepts addressed (i.e., importance, ability/difficulty, change over time) (see Table 1). Questions were answered as Yes/No (e.g., Was this question easy to understand? Were the examples helpful? Is there a need for a tool like the JDAPT?). Each question was followed with an open-ended response option for the respondent to explain their answer and provide details. The interviews took about 60–90 min to complete. They were conducted between October 2019 to April 2020, with most conducted before the first COVID-19 lockdown in Canada.

**Table 1:** Topics assessed for sensibility of the job demands and accommodation planning tool (JDAPT)

Screening questionnaire:
1. Understandability: Instructions clear; examples improve question clarity; domain labels make sense/are clear
2. Relevance: Questions got me thinking about this job’s demands; would continue with the complete JDAPT questions after the screener
3. Feasibility: Easy to complete the domain questions
4. Length: Recorded time to complete
5. Overall impressions: Includes open-ended question responses
Full JDAPT questionnaire:
1. Comprehensiveness: Domain questions perceived as complete (no additions recommended)
2. Understandability: Instructions clear; Made sense to ask about importance of demand to job, job ability/difficulties, changes in ability over time; domain labels make sense/are clear
3. Relevance: Questions helped me to think about my/the job; the JDAPT would help someone understand a worker’s challenges, there is a need for the JDAPT; would use the JDAPT
4. Feasibility: Easy to complete overall; Detailed job demands questions easy to answer (importance of demand to job, ability/difficulty performing, changes in ability over time)
5. Length: Recorded time to complete; perceptions of length
6. Overall impressions: Includes open-ended question responses

### Analyses and Performance Criteria

Frequencies for each question were compiled. There exist few performance guidelines for sensibility assessments [58–60]. However, similar to other research, we adopted a 70% participant endorsement rate as the minimum level of acceptability for each of the sensibility concepts [58] with a higher percentage reflecting greater perceived comprehensiveness, understandability, relevance, feasibility, and acceptance of the length of the questionnaire. Open-ended responses were content analyzed for major themes by two authors (JB & ST) [66]. Participant suggestions for new examples and items were collected and reviewed by MAMG, JB, and ST prior to discussion with the research team. The concordance of the screening responses with the full JDAPT questionnaire also was examined. We focused on respondents’ assessments of their global ability/difficulty for each of the four broad domains (physical, cognitive, working with others, working conditions) in the screening questions and whether it corresponded with their assessments of ability/difficulty once they had completed the specific job demands questions from each domain.

## Results

Forty-six workers living with a chronic condition causing an episodic disability and 23 organizational stakeholders (total n = 69) participated in the study (see Table 2). Most participants were women (78.3%). We recruited a range of ages among workers and organizational representatives. Most participants had some post-secondary education (89.9%). Among workers, 71.7% lived with a chronic physical condition causing an episodic disability, 13.0% lived with a mental health condition and 15.2% lived with both a physical and a mental health condition. Examples of conditions reported by participants include rheumatoid arthritis, epilepsy, multiple sclerosis, depression, anxiety disorders, fibromyalgia, Crohn’s disease, vertigo, bipolar disease, chronic fatigue syndrome, mild traumatic brain injury and ulcerative colitis. Most organizational representatives did not report having a chronic health condition (60.9%), although 13.0% reported a physical condition, 17.4% reported a mental health condition, and 8.7% reported having both.

**Table 2:** Sample characteristics (n = 69)

Characteristic	Workers	Organizational respondents	All
	n = 46	n = 23	n = 69
	Mean (SD)/n (%)	Mean (SD)/n (%)	Mean (SD) /n (%)
Gender (women)	37 (80.4)	17 (73.9)	54 (78.3)
Age (years)			
18–34	20 (43.5)	4 (17.4)	24 (34.8)
35–49	18 (39.1)	11 (47.8)	29 (42.0)
≥ 50	8 (17.4)	8 (34.8)	16 (23.2)
Post-secondary education	41 (89.1)	21 (91.3)	62 (89.9)
Chronic condition type			
Physical	33 (71.7)	3 (13.0)	36 (52.2)
Mental/Cognitive	6 (13.0)	4 (17.4)	10 (14.5)
Both	7 (15.2)	2 (8.7)	9 (13.0)
No chronic condition	–	14 (60.9)	14 (20.3)
Employment sector			
Financial, technology, government, insurance	12 (26.1)	2 (8.7)	14 (20.3)
Education, health, sciences arts, professional	25 (54.3)	15 (65.2)	40 (57.9)
Sales, services, retail	3 (6.5)	5 (21.7)	8 (11.6)
Construction, agriculture, manufacturing, utilities	6 (13.0)	1 (4.3)	7 (10.1)
Organization size			
< 50 people	7 (15.9)	5 (22.7)	12 (18.2)
50–499 people	6 (13.6)	8 (36.4)	14 (21.2)
≥ 500 people	31 (70.5)	9 (40.9)	40 (60.6)
Full-time (≥ 35 h/week)	34 (73.9)	22 (95.7)	56 (81.2)
Contract work	12 (26.1)	3 (13.0)	15 (21.7)
Job tenure (years)	6.7 (6.8)	7.6 (7.0)	7.0 (6.8)
Supervisory responsibilities	11 (23.9)	18 (78.3)	29 (42.0)

n’s can vary due to missing data

Participants worked in diverse sectors with about half of workers employed in either education, health, sciences, arts, or professional jobs. Example job types among workers included being a transport and delivery driver, baker, nurse, accounts manager, teacher, administrative assistant, software engineer, clinical researcher, and client manager. About 70% of workers were employed in large organizations of over 500 employees compared to 40.9% of organizational representatives. Most workers were employed full-time (73.9%), 23.9% had supervisory responsibilities, and 26.1% were contract workers. Not surprisingly, more organizational representatives worked full-time (95.7%), had supervisory responsibilities (78.3%), with fewer reporting contract work (13.0%) (see Table 2).

### Screening Questions

Table 3 presents the findings for the screening questions. In all cases, worker and organizational respondents’ endorsements exceeded the 70% threshold for understandability, relevance, and feasibility of the questions. Endorsement levels for worker and organizational respondents were relatively similar and often exceeded 85%. Nearly all participants reported that the instructions were clear and that examples were helpful (all endorsements over 93%). Over 70% of respondents rated the domain labels as clear, especially with the use of examples to explain what was meant by cognitive or “thinking” tasks and working with others. Workers rated the relevance of questions in helping them to think about their job and in willingness to continue completing the JDAPT as greater than 95%. This was somewhat greater than for organizational representatives, although this group rated the relevance of the screening questions at 87%, which highly exceeds the minimum performance criteria. On average, worker and organizational representatives took between 3–4 min to complete the screening questions, with workers taking a little longer than organizational respondents.

**Table 3:** Screening questionnaire sensibility and congruence with complete job demands and accommodation planning tool (JDAPT)

Sensibility characteristic	Workers	Organizational respondents	All
	n = 46	n = 23	n = 69
	n (%)	n (%)	n (%)
Understandability			
Instructions clear	45 (97.8)	22 (95.7)	67 (97.1)
Examples help clarify			
Physical demands	46 (100)	23 (100)	69 (100)
Cognitive demands	44 (95.7)	22 (95.7)	66 (95.7)
Working with others	43 (93.5)	23 (100)	66 (95.7)
Working conditions	44 (95.7)	22 (95.7)	66 (95.7)
Domain labels are clear	35 (76.1)	17 (73.9)	52 (75.4)
Relevance			
Questions helped me think about my/the job	44 (95.7)	20 (87.0)	64 (92.8)
Would continue completing the JDAPT	44 (95.7)	22 (87.0)	66 (95.7)
Feasibility			
Easy to complete			
Physical demands	38 (82.6)	22 (95.7)	60 (87.0)
Cognitive demands	40 (87.0)	22 (95.7)	62 (89.9)
Working with others	41 (89.1)	18 (78.3)	59 (85.5)
Working conditions	43 (93.5)	22 (95.7)	65 (94.2)
Length			
Minutes to complete (mean/SD)	3.7 (1.7)	3.0 (1.4)	3.5 (1.7)
Congruence of Screening questions and complete JDAPT			
Ability/Difficulty questions			
Physical demands	33 (71.2)	19 (82.6)	52 (75.4)
Cognitive demands	35 (76.1)	22 (95.6)	57 (82.6)
Working with others	30 (60.5)	16 (69.6)	46 (66.7)
Working conditions	28 (60.9)	19 (82.6)	47 (68.1)

Despite high levels of endorsement provided for the sensibility concepts in the screening questions, a comparison of the screening questions with the complete JDAPT questionnaire found that congruence between them was problematic (see Table 3). Specifically, when asked about their ability/difficulty with each of the four domains on the screening questions, many respondents reported no difficulties. However, when completing the full JDAPT and the more specific questions within a domain, respondents sometimes changed their answers and reported at least some difficulty with at least one job demand within that domain. Rarely did a respondent report difficulty with a domain in the screening questions but no difficulty after reviewing the detailed job demands. Discordance was more common among worker respondents than organizational respondents and was noted for the domains working with others and working conditions. For example, when asked in the screening questionnaire about their overall ability/difficulty in working with others, 60.5% of workers with chronic conditions provided answers that were congruent with their answers to the four specific job demands comprising working with others in the complete JDAPT. However, nearly 40% (39.5%) of workers with chronic conditions reported no difficulties with working with others in the screening questionnaire, but upon answering the individual items, reported at least some difficulty with at least one job demand in this domain. Across the four domains, worker congruence ranged from 60.5 (working with others) to 76.1% (cognitive demands) leaving approximately 25 to 40% of respondents reporting discordance between the screening questions and complete JDAPT. Organizational respondent congruence ranged from 69.6 (working with others) to 95.6% (cognitive demands). Overall, 47 (68.1%) worker or organizational participants had some discordance between the screening questions and the complete JDAPT.

### Complete JDAPT Questions

Table 4 presents the results for the sensibility of the complete JDAPT questions. Responses reached or exceeded the performance criteria for comprehensiveness of domain questions for three of the four job demand domains. Endorsement thresholds were below 70% for working with others (worker = 63.0%; organizational = 65.2%). An examination of the open-ended responses for all domains found several trends. First, several respondents added items that were already encompassed by a job demand but that were more nuanced and detailed. This could be addressed by adding examples to an existing job demand. For example, one respondent added “pushing and pulling objects” to their physical job demands. Instead of creating a new job demand, we added this as an exemplar to an existing job demand of work activities that required strength. Respondents also sometimes suggested adding aspects of work like commuting to and from their job which was not part of their job demands but because it was difficult for them. The JDAPT focuses only on aspects of a person’s required job demands where an employer might be asked to provide support or accommodations. As a result, work-related travel (e.g., visiting clients, visiting job sites, traveling to meetings) was included as a job demand, but general commuting to and from work was not included. Similarly, within the domain of working with others, where participants made the most suggestions for additions, comments were largely about getting along (or not) with colleagues and perceptions of support. Although interpersonal dynamics are an important component of an organization’s climate, for the most part the support appraisals provided by respondents did not constitute a required part of workers’ job activities. In other cases, it was judged that suggestions were already captured by existing JDAPT questions related to working with others.

**Table 4:** Sensibility of the complete job demands and accommodation planning tool (JDAPT) questions

Sensibility characteristic	Workers	Organizational Respondents	All
	n = 46	n = 23	n = 69
	n (%)	n (%)	n (%)
Comprehensiveness			
Domain questions complete (no new job demands added)			
Physical demands	33 (71.7)	17 (73.9)	50 (72.5)
Cognitive demands	34 (73.9)	18 (78.3)	52 (75.4)
Working with others	29 (63.0)	15 (65.2)	44 (63.8)
Working conditions	37 (80.4)	20 (87.0)	57 (82.6)
Understandability			
Instructions clear	45 (97.8)	22 (95.7)	67 (97.1)
Made sense to ask about…			
Importance of demand to job	46 (100)	21 (91.3)	67 (97.1)
Ability/difficulty performing	44 (95.7)	20 (87.0)	64 (92.8)
Changes in ability over time	44 (95.7)	19 (82.6)	63 (91.3)
Domain labels are clear	35 (76.1)	17 (73.9)	52 (75.4)
Relevance			
Questions helped me think about my/the job	46 (100)	21 (91.3)	67 (97.1)
Would help someone understand a worker’s challenges	43 (93.5)	21 (91.3)	64 (92.8)
Perceived need for JDAPT	43 (93.5)	21 (91.3)	64 (92.8)
Would use JDAPT	41 (89.1)	20 (87.0)	61 (88.4)
Feasibility			
Easy to complete overall	41 (89.1)	18 (78.3)	59 (85.5)
Detailed job demand questions easy to answer			
Importance of demand to job	40 (87.0)	19 (82.6)	59 (85.5)
Ability/difficulty performing	32 (69.6)	16 (69.6)	48 (69.6)
Changes in ability over time	35 (76.1)	17 (73.9)	52 (75.4)
Length			
Minutes to complete (mean/SD)	15.1 (6.1)	12.4 (5.0)	14.2 (5.9)
Length perceived as adequate	35 (76.1)	18 (78.2)	53 (76.8)

After reviewing the comments, three new job demands were created to enhance comprehensiveness. For physical job demands, we added a question asking whether using one or more of a person’s senses (i.e., touch, taste, smell, hearing, and seeing) was important to the job and provided examples related to each sense like food tasting and distinguishing fine visual details. For working with others, we added a job demand asking about participating in social activities and meeting social expectations with examples like networking, attending after hours events, and volunteering for workplace committees. Finally, for working conditions, we added a job demand that asked participants whether they had to work in locations where they did not have easy access to facilities that would meet their personal needs, such as having no easy access to a washroom, no running water, or access to a refrigerator for food or medication.

Endorsements for understandability of the complete JDAPT was excellent with responses from workers and organizational representatives often exceeding 85% (see Table 4). This included asking questions about the understandability of response keys that assessed the importance of a job demand, ability/difficulty with the demand, and changes over time. Endorsement of domain labels exceeded 70% but were somewhat lower (worker = 76.1%; organizational representatives = 73.9%). Comments from participants noted that terms like “cognitive or thinking demands,” “working with others” and “working conditions” weren’t always immediately clear. However, respondents consistently reported that examples clarified the labels, and that they were able to understand the areas of their job to which the labels referred.

Endorsements of the relevance of the JDAPT also were excellent and often approached or exceeded 90%. Workers and organizational representatives reported that the JDAPT helped them to think about the job, understand a worker’s challenges and perceived need, and that they would use the JDAPT (see Table 4). For example, a worker living with a mental health condition stated:

I go in and just say, “I’m dealing with a mental illness” and my boss says to me, “what do you need?” And I say, “I don’t know”—which is what happened. I think [the JDAPT] might be better to help me understand actually, that these three aspects of the job are what are actually difficult for me, so let’s think about what we do with those. (Worker: Policy advisor living with depression and PTSD; government)

An organizational representative who was a manager in a hospital echoed this perspective, noting “[The JDAPT] forces you to think about all the ways that a person could have difficulty that you wouldn’t necessarily think about.”

Feasibility of the complete JDAPT varied. Endorsement of the overall ease of completion was 89.1% in workers and 78.3% in organizational participants. Endorsements for the ease of completion of questions about the importance of a job demand were excellent and exceeded 80%. However, assessments of ease of completion of questions about the ability/difficulty questions were 69.6% for both workers and organizational representatives. Questions about changes in ability over time were endorsed by 76.1% of workers and 73.9% of organizational representatives. Open-ended responses explaining the lower endorsements indicated that, although workers understood the questions, answering about ability/difficulty and changes over time when one lives with an episodic disability can take time to consider. Arriving at an answer was do-able, but not “easy.” This did not diminish the perceived importance of the tool. Time taken to consider one’s answer was viewed as a necessary step that enhanced the validity of the JDAPT. Similarly, organizational representatives reported that the questions were easy to understand. However, in considering their answer, they reported not always being certain whether a worker’s difficulties or changes in performance were related to their health condition or some other aspect of their skills or motivation. Yet, despite not always being sure about the attribution of a problem to a worker’s health, organizational participants were confident that there was a difficulty with the performance of a job demand.

On average, participants took 12–15 min to complete the JDAPT. Endorsements of the length as acceptable were 76.1% of workers with a chronic condition and 78.2% of organizational representatives. Comments providing an overall assessment of the JDAPT highlighted that the questions were clear and easy to understand and covered the relevant job demands (see Table 5). Participants also reported that the examples and the use of a clear pattern to lay out the job demands followed by the importance to the job, any difficulties, and any changes over time aided them in understanding and responding to the items. Questions were assessed as thought provoking and ensured that a person considered specific demands of the job.

**Table 5:** Worker and organizational representative comments assessing the job demands and accommodation planning tool (JDAPT)

Comprehensiveness:	“It seemed like you covered every piece, whether it was the physical piece, tedious work that’s happening over and over again, or working long hours, or travelling—so you have covered everything.” (Worker: baker living with ADHD and a skin condition)
	“There were basically all of them that I go through in my job. It’s basically everything that I do at work.” (Worker: truck driver living with Crohn’s disease)
	“I thought that you did a very good job of breaking down each of the different categories into sort of sub-categories. Like your questions on physical demands, for example, covered almost every physical demand I could think of. Same goes for cognitive demands and the other categories as well. I thought that was really great.” (Worker: health and safety specialist living with irritable bowel syndrome and anxiety)
Understandability & Feasibility:	“It was helpful. It broke it down into good categories, gave good explanations. And then, it was very uniform, so when I completed one section and then I moved to the next, it was basically asking me the same thing, so I didn’t have to re-read everything again… So it was easier to follow, because you were kind of, not anticipating, but that’s maybe the best word I can use.” (Worker: adjudicator living with multiple sclerosis)
	“They were really clear. The examples were really helpful. I found that because the question [pattern was] the same every time, I found it really easy to fill out.” (Worker: clinical research coordinator living with multiple physical conditions)
	“I think it’s pretty easy to use, and I think it would be useful, especially [because it’s] broken down into physical, cognitive demands, and so on. It’s an easy tool to use, and it’s beneficial.” (Organizational representative: supervisor, hospitality services)
Relevance:	“[It] makes you think about your job and what you’re doing, so it’s reflective.” (Worker: librarian living with multiple sclerosis)
	“It’s giving you information directly about the direct areas that you struggle with the most at work. And secondly, it could be a jumping off point to talk to an employer about what they might be able to do or how they could help to modify the job.” (Worker: support teacher living with fibromyalgia)
	“The way that you’ve separated [the questions] into those four major domains—I think it forces you to think about any job that you’re considering…from multiple angles, and not just in the context of a generalized job description. I think separating them into different areas and different ways a person can be impacted in their job is really helpful. It forces me to think about it in a more detailed way than I otherwise would have." (Organizational representative: manger, healthcare)
	“It definitely gets you thinking.” (Organizational representative: human resources manager, retail)
Length:	“Initially when I was going through the questions, oh, wow, it might take some time. But then once you start doing each question, it’s not as demanding or intimidating. Because that’s something to be mindful about, that a lot of us get mental fatigue and I feel the questionnaire was respectful of that aspect, so no concerns from that perspective.” (Worker: accountant living with multiple sclerosis)
	“It’s pretty detailed but I think it needed to be that length…I think you would probably lose some good information if you shortened it." (Worker: policy advisor living with arthritis)
	“It seemed intimidating at first when I saw 23 pages, but it seemed to go by very easily. It just seemed to be six questions per section.” (Organizational representative: supervisor, customer service)
	“I think the person will need to invest some time in this to make sure that things are being filled out properly. I think it is a little bit long, but I do understand that you kind of need to invest this time to do this kind of an evaluation.” (Organizational representative: human resources specialist, financial sector)

## Discussion

Guidance on ways to communicate workplace support and accommodation needs is an important priority among workers living with chronic health conditions. This study examined the sensibility of the JDAPT questions, focusing on comprehensiveness, understandability, relevance, feasibility, and length from the perspective of workers living with chronic conditions causing episodic disabilities, and organizational representatives who often provide support to these workers. Sensibility testing allows researchers to assess early development issues in a tool prior to additional psychometric testing and outcome evaluation. The JDAPT was created to fill an important gap related to the ease with which workers and organizational representatives could appraise a job’s demands and identify areas where support might be beneficial. The study yielded similar findings from women and men across different ages, health conditions and job types. The findings highlighted high endorsement of the JDAPTs comprehensiveness and feasibility, although study participants noted additional job examples and demands that could enhance the tool. Perceived relevance of the JDAPT was excellent and highly exceeded our minimum threshold of endorsement, pointing to the need for and potential usefulness of the tool. Research examining sensibility is sometimes overlooked in initial testing of a tool but is critical to ensure inclusivity and that the perspectives of users are paramount in real-world tool development and evaluation. This study provides a first step in that assessment.

An important aspect of sensibility testing is to engage potential users of a tool and draw on their insights and experiences. Our cognitive interviewing included people working with physical and mental health conditions, as well as organizational representatives with experiences supporting employees with episodic disability, some of whom also lived with a chronic health condition. Many previous workplace studies have focused exclusively on a particular type of condition (e.g., rheumatoid arthritis), have not included both physical and mental health conditions, and have not included workplace representatives who provide support to workers. We also drew participants from across industrial sectors, work arrangements, and organizational sizes, included participants from smaller workplaces, and part-time and contract workers. The diversity of our sample was helpful not only in the sensibility assessments, but also in enhancing the examples we were able to add to JDAPT questions and in identifying new job demands that have not typically been included as important aspects of employment in other studies. Across groups and sensibility constructs, JDAPT endorsements were high pointing to the potential wide applicability of the tool. At the same time, our sample was convenience-based. For example, we lacked representation from primary industries (e.g., agriculture/farming, mining, logging/forestry). Our participants also had high levels of education. Among organizational representatives, this is not surprising as supervisory and leadership roles often call for greater education. Among workers living with chronic health conditions, educational patterns may be changing. There is emerging evidence that younger adults living with episodic disabilities often pursue college and university education as a means of securing jobs with better benefits and a greater likelihood of accommodation availability [67, 68]. Unfortunately, data also suggest that many individuals living with a disability report underemployment where their jobs do not fully make use of their skills [69, 70]. Canada has relatively high levels of education with Statistics Canada finding that 86.3% of Canadians have a high school diploma or its equivalent and that 54% of Canadians have college or university certifications [71]. Despite increasing educational levels, additional testing with the JDAPT is needed to include individuals with less education and with English as a second language, as these factors are linked to the nature of the job demands undertaken by individuals. It is particularly important for assessment of the understandability and feasibility of the JDAPT.

As part of the JDAPT, we created a screening questionnaire with the goal of enhancing the feasibility of the tool and allowing a shortened version to be completed by participants who were not experiencing any difficulties within a broad job domain. Although the screening questions were highly understandable, relevant, easy, and quick to answer, the discordance between the screener and complete JDAPT questions suggested that, going forward, the screening questions should be omitted from the tool and that all participants complete the full JDAPT questions. Given that the length of the complete JDAPT was endorsed as acceptable, completing the full JDAPT should not be too onerous for most respondents. The lack of concordance also highlighted some positive elements of the complete JDAPT. Specifically, it provided initial evidence that, although many individuals may believe they have a good understanding of the challenges within a job, they may omit, or underestimate difficulties related to the work unless a more in-depth assessment of different job demands is undertaken. This was especially true when it came to thinking through the interpersonal aspects of a job and a job’s working conditions. These domains have not always received the same attention as the physical demands of a job, although recent research has highlighted the complexities of providing accommodations related to interpersonal aspects of working [12]. By asking diverse questions about job demands related to working with others and working conditions, workers and organizational representatives were able to better assess a full range of challenges with the job. Ultimately, this may translate into a more comprehensive assessment of support and accommodation needs.

As noted, the cognitive interviews enabled participants to draw from their experiences and enhance the examples and types of job demands included in the JDAPT. As a result of participant input, three new job demands were added. These were: whether the use of one or more of one’s senses was an important part of the job (e.g., being a taster in a kitchen), whether the job included social demands or meeting social expectations (e.g., attending after hours events, networking, committee volunteering), and whether the job required working in locations where access to facilities to meet personal needs was difficult (e.g., working in a kiosk in a shopping mall with no access to a refrigerator for medication). The addition of these types of job demands warrant additional attention in future research. They also challenge organizations to more comprehensively think about supports and accommodations that may be needed to make employment more accessible and inclusive to all workers.

There is no definitive approach or gold standard for assessing sensibility [54, 55, 57–59]. We used a 70% endorsement as our standard, which has been adopted elsewhere [58]. Ratings often highly exceeded this cut-off with overall ratings from workers’ endorsements of the JDAPT’s relevance ranging from 89 to 100% for willingness to use the JDAPT, perceived need, helping others understand a worker’s challenges, and thinking about their job. At the same time, responses indicated that appraising one’s ability/difficulty with work tasks, and assessing changes over time, was not always easy. Some participants, especially those with a highly variable chronic condition, needed time to think about their work and health needs so that they felt comfortable providing an answer that they believed captured their situation. Responses to open-ended questions suggested that participants believed the questions were helpful in thinking about their health in new ways and in better understanding the links between specific job demands and areas where support might make a difference. Many had not thought about their health condition in this way previously. At the same time, additional testing, especially longitudinal research measuring perceptions of job abilities and changes over time is needed, as well as outcome evaluation research that examines the efficacy of support and accommodations for different types of job difficulties.

Organizational representatives also rated the JDAPT as highly relevant with endorsements ranging from 87 to 91%. A key issue among organizational participants was the extent to which they had been given information by workers about a chronic health condition’s impact that they were then able to draw upon when completing the JDAPT. Responses to the open-ended questions suggested that organizational representatives were not always certain about the attribution of difficulties to a worker’s health, and whether other factors contributed to difficulties like a lack of skills or absence of motivation. Despite this, they were confident in recognizing that difficulties existed with a job demand. Further research with organizational representatives is needed. For example, it would be helpful to assess the congruence of JDAPT responses if completed by a worker for their health and an organizational representative like a worker’s supervisor. Research also would be beneficial to examine standard practices within organizations, which currently range from informal discussions to more comprehensive functional and cognitive assessments, and whether the JDAPT provides an equally comprehensive assessment of support needs and solutions.

There are several strengths and limitations to this research. Strengths include that the JDAPT goes beyond existing research tools and aims to provide a way for workers to assess their intermittent and ongoing job demands and support needs, and to receive a range of ideas and strategies to better manage challenges working. Strengths also include the inclusion of a diverse sample of workers and organizational representatives who drew on their personal experiences of working with (or supporting an employee with) a chronic condition causing an episodic disability. Our methods targeted several aspects of sensibility testing, established criteria for endorsement, and provided multiple opportunities for respondents to comment on the JDAPT in their own words. Limitations include that our sample was recruited using convenience-based methods and was cross-sectional in design. Future research needs to include a larger and more diverse group of respondents, including more men, individuals with lower levels of education and English as a second language, as well as longitudinal data to assess changes in difficulty over time. Efforts also need to be made to include more racial and ethnic diversity and to examine new immigrant worker needs in diverse occupations. Larger samples would reduce the potential for bias and would enable sub-group analyses. Because this study focused on sensibility perceptions from potential users of the JDAPT, it did not include a comprehensive evaluation of support and accommodation ideas that would be provided by the JDAPT.

Evaluation next steps for the JDAPT also include outcome and effectiveness evaluation to assess the validity and effects of the JDAPT in real-world conditions [72]. This evaluation process, sometimes labeled a *holistic effectuality evaluation approach* recognizes that real-world outcome evaluations are a hybrid containing both constructive and conclusive evaluation elements [72]. That is, implementation, adoption and usefulness of a tool will be influenced by external factors such as culture, workplace norms, and social support, and may also relate to personal factors like age, gender, and type of episodic condition. Going forward, evaluation needs to consider these elements as it examines the uptake of the JDAPT. Evaluation efforts also should examine whether there is meaningful change over time in worker and organizational representative perceptions and behaviours. Outcomes should include understanding whether use of the JDAPT is associated with behaviour changes at work like adoption of different support strategies and perceptions of the JDAPTs usefulness, whether there is ongoing use of the tool over time when a worker’s health or job needs change, and whether there are changes in work outcomes like improved productivity and job sustainability.

## Conclusions

This study examined sensibility perceptions of a new tool, the JDAPT, among workers living with chronic physical and/or mental health conditions, as well as organizational representatives who provide supports to workers. Increasing numbers of people are working with chronic health conditions that cause episodic disability. They can find it challenging to identify and discuss workplace support and accommodation needs. Study findings highlight the need and relevance of the JDAPT and that it identifies job demands that often are not assessed in other tools, as well as being comprehensive, understandable, and relatively easy to complete. Improving the support provided to people living with chronic physical and mental health conditions is critical to create more inclusive employment opportunities and sustain long-term employment goals.

## Appendix A

See Table 6.

**Table 6:** Types of job demands assessed across physical, cognitive, interpersonal, and working conditions in the job demands and accommodation planning tool (JDAPT)

Physical demands
• Moving around, or working in awkward positions or postures
• Working with your hands
• Job activities related to strength
• Physical endurance or stamina
• Using one or more of your senses (touching, tasting, smelling, hearing, seeing) [added after testing]
Cognitive demands (i.e., mental or “thinking” job demands)
• Paying attention to detail or remembering information
• Concentrating for long periods of time
• Responding to changing work demands
• Problem solving or critical thinking
• Multi-tasking
• Managing time pressures
Interpersonal demands (i.e., working with others)
• Working with others
• Supervising others
• Communicating, negotiating or motivating others
• Dealing with the emotions of other people
• Social activities or meeting social expectations [added after testing]
Working conditions (i.e., conditions of work and work environment)
• Working around distractions
• Working in extremes of temperature, weather or other conditions
• Working with hazardous equipment or hazardous conditions
• Isolated work
• Working or being at work during specific times
• Work-related travel
• Working in situations where making an error could have critical consequences
• Working in locations where you do not have easy access to facilities to meet your personal needs [added after testing]
